# Urgences hypertensives au Centre hospitalier universitaire de Bogodogo, Ouagadougou (Burkina Faso)

**DOI:** 10.48327/mtsi.v4i3.2024.500

**Published:** 2024-09-03

**Authors:** Wendlassida Martin NACANABO, Taryètba André Arthur SEGHDA, Djième Claudine DAH, Wendlassida Léa Françoise SAWADOGO, Issa SAWADOGO, Mohamed Saidou DIMZOURÉ, Murielle LOYA, Lamoundi Prisca THIOMBIANO, André Koudnoaga SAMADOULOUGOU

**Affiliations:** Service de cardiologie du Centre hospitalier universitaire de Bogodogo, Ouagadougou, Burkina Faso

**Keywords:** Urgences hypertensives, antihypertenseurs, Ouagadougou, Burkina Faso, Afrique subsaharienne, Hypertensive emergencies, antihypertensive drugs, Ouagadougou, Burkina Faso, Sub-Saharan Africa

## Abstract

**Introduction:**

Les urgences hypertensives sont définies comme une poussée hypertensive avec signes de souffrance viscérale. Elles sont relativement fréquentes et nécessitent une thérapeutique antihypertensive urgente, mais pas obligatoirement normotensive. L'objectif de cette étude est de décrire l’épidémiologie des urgences hypertensives.

**Patients et méthodes:**

Il s'est agi d'une étude transversale à visée descriptive qui s'est déroulée du 5 mars 2017 au 31 décembre 2023 dans le service de cardiologie du Centre hospitalier universitaire de Bogodogo (CHU-B), Burkina Faso. Ont été inclus dans l’étude tous les patients admis dans le service pour urgences hypertensives. Les caractéristiques épidémiologiques, socio-démographiques, cliniques, paracliniques, thérapeutiques et évolutives ont été évaluées en analyse descriptive.

**Résultats:**

Parmi les 2 610 patients hospitalisés, 96 cas d'urgences hypertensives ont été colligés, soit une prévalence de 3,6 %. L’âge moyen était 50,5 (±10,4) ans. Les facteurs de risque cardiovasculaire retrouvés étaient l'hypertension artérielle, la sédentarité, le diabète et le tabac, respectivement dans 80,8 %, 72,3 %, 12,7 % et 9,5 % des cas. Les motifs de consultation étaient les céphalées (46,8 % des cas), suivies des vertiges (19,1 %). L'accident vasculaire cérébral ischémique et l’œdème aigu du poumon étaient observés dans 18,7% des cas.

**Conclusion:**

Les urgences hypertensives sont assez fréquentes à l'hôpital du CHU-B, et la majorité des patients est relativement jeune.

## Abstract (en)

**Introduction:**

Hypertensive emergencies are defined as hypertensive crisis with signs of visceral distress. They are relatively common and require urgent, but not necessarily normotensive, antihypertensive therapy. The aim of this study was to describe the epidemiology of hypertensive emergencies..

**Patients and methods:**

This was a descriptive cross-sectional study conducted from March 5, 2017, to December 31, 2023, at the cardiology department of the Bogodogo University Hospital (CHU-B), Burkina Faso. All patients admitted to the department for hypertensive emergencies were included in the study. Epidemiologic, sociodemographic, clinical, paraclinical, therapeutic, and evolutionary characteristics were evaluated in a descriptive analysis.

**Results:**

Among 2,610 hospitalized patients, 96 cases of hypertensive emergencies were identified, representing a prevalence of 3.6%. Mean age was 50.5 (±10.4) years. Cardiovascular risk factors were hypertension, sedentary lifestyle, diabetes, and smoking in 80.8%, 72.3%, 12.7%, and 9.5% of cases, respectively. Headache was the most common reason for consultation (46.8% of cases), followed by dizziness (19.1%). Ischemic stroke and acute pulmonary edema were observed in 18.7% of cases.

**Conclusion:**

Hypertensive emergencies are quite frequent at CHU-B and the majority of patients are relatively young.

## Introduction

L'urgence hypertensive (UH) est définie par une élévation brutale de la tension artérielle atteignant ou dépassant 180/110 mmHg avec signes de souffrance viscérale [18, 19]. Elle nécessite une thérapeutique antihypertensive, mais pas obligatoirement normotensive [2]. Quant à la « fausse urgence hypertensive » ou urgence relative, elle se définit comme une élévation de la tension artérielle isolée sans souffrance d'organe [12]. Ces poussées hypertensives sévères sont relativement rares et concernent 1 à 2 % des patients hypertendus [8, 12]. Toute l'urgence repose sur la mise en évidence de l'atteinte aiguë d'un organe cible qui nécessiterait une hospitalisation immédiate dans un service adapté [8]. Au Burkina Faso, il n'y a pas d’étude sur toutes les formes d'urgences hypertensives. Les seules études réalisées concernent l'hypertension artérielle maligne et la super hypertension artérielle avec respectivement des prévalences hospitalières de 18,4 % et de 12,9 % des patients hypertendus [13, 20]. L'objectif de ce travail est d’étudier les aspects épidémiologiques, cliniques, paracliniques et thérapeutiques des urgences hypertensives dans le service de cardiologie du Centre hospitalier universitaire de Bogodogo (CHU-B).

## Patients et méthode

Il s'est agi d'une étude transversale à visée descriptive qui s'est déroulée du 5 mars 2017 au 31 décembre 2023 dans le service de cardiologie du CHU-B. Ont été inclus dans l’étude les patients admis pour urgence hypertensive. Les variables collectées étaient :

- les données sociodémographiques et cliniques prenant en compte les facteurs de risque cardiovasculaire, l’état hémodynamique et les signes de souffrance viscérale;
- les paramètres électrocardiographiques (hypertrophie des cavités cardiaques, présence d'une ischémie-lésion-nécrose, trouble du rythme ou de la conduction);
- les paramètres écho-cardiographiques et Doppler (état des cavités droites, dilatées ou non, présence de thrombus, fonction systolique du ventricule gauche, altérée ou non);
- les paramètres scanographiques (type d'atteinte cérébrale vasculaire et sa localisation);
- l'atteinte rétinienne classée en trois stades selon Kirkendall [7];
- les paramètres biologiques incluant la protéinurie, la glycémie, la troponine, les lipides, la créatininémie et l'azotémie.

La saisie et l'analyse statistique des données collectées ont été réalisées grâce au logiciel Epi-info. La description des différentes variables a été effectuée avec le calcul des proportions pour les variables qualitatives, et les paramètres de position et de dispersion pour les variables quantitatives.

## Résultats

**Figure 1:**
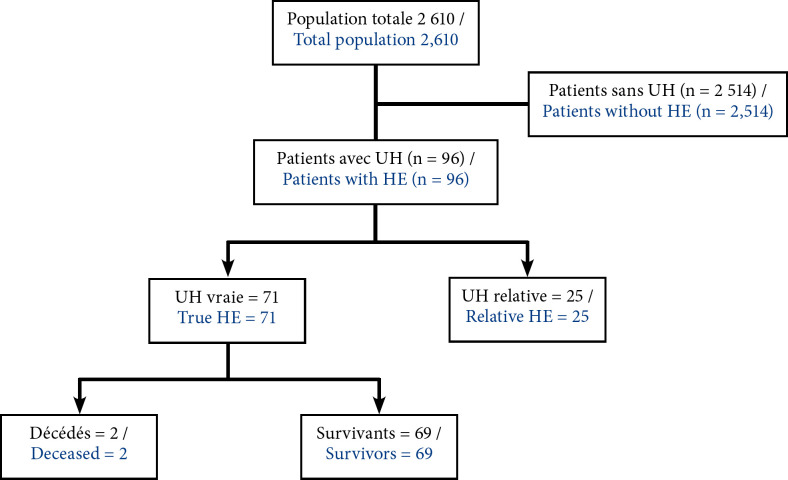
Diagramme de flux des patients

Parmi les 2 610 patients hospitalisés dans le service de cardiologie toutes causes confondues, on notait 96 cas d'urgences hypertensives soit une fréquence hospitalière de 3,6 %. Le diagramme des flux de notre étude est représenté par la Figure 1. L’âge médian des patients était de 50 ans (premier quartile 38 ans et troisième quartile 60 ans) avec des extrêmes allant de 23 à 83 ans. On notait une prédominance féminine avec 55 femmes (58,5 %) contre 39 hommes (41,5 %). Soixante-seize de nos patients, soit 80,8 %, avaient un antécédent d'hypertension artérielle. Les motifs de consultation étaient les céphalées, les vertiges et le déficit moteur, retrouvés respectivement chez 44, 18 et 15 patients (Tableau I).

**Tableau I:** Caractéristiques générales des patients au CHU-B

Variables	Effectifs (n)	Proportions (%)
Facteurs de risques cardiovasculaires et comorbidités		
Hypertension artérielle	76	80,8
Sédentarité	68	72,3
Diabète	12	12,7
Tabac/ Tobacco	9	9,5
Hypertension artérielle familiale	7	7,4
Maladie rénale chronique	5	5,3
Motifs de consultations		
Céphalées	44	46,8
Vertiges	18	19,1
Déficit moteur	15	15,9
Acouphènes/phosphènes	9	9,5
Aspect évolutifs		
Médiane de la durée d'hospitalisation en jours	05	[1;5]
Tension artérielle contrôlée à la sortie	48	51
Décédés	2	2

**Figure 2:**
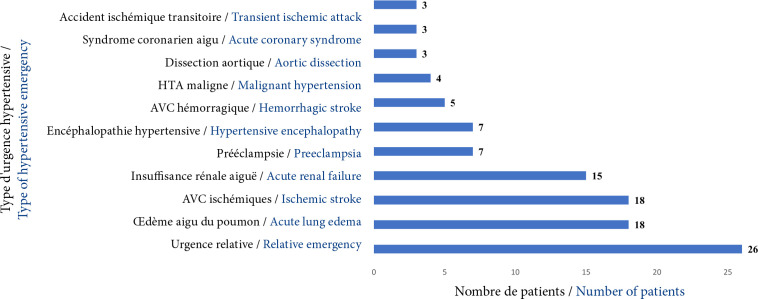
Représentation graphique des patients en fonction du type d'urgence hypertensive au CHU-B

Les urgences hypertensives vraies concernaient 73,4 % (n=71) des patients. Parmi les urgences vraies, étaient retrouvés l'accident vasculaire cérébral ischémique dans 18,7% (n=18) des cas, l’œdème aigu du poumon et l'insuffisance rénale aiguë dans 18,7 % (n=18) et 15,6 % (n=15) des cas respectivement. Un quart du groupe étudié présentait une urgence relative. Les différents types d'urgences hypertensives sont résumés dans la Figure 2.

L’électrocardiogramme montrait une hypertrophie auriculaire et ventriculaire gauche dans respectivement 41,5 % et 36,2 % des cas. Quatre patients avaient un hémibloc antérieur et une fibrillation auriculaire était observée chez cinq patients. À l’échocardiographie transthoracique, 12,8 % (n=12) avaient une dilatation des cavités gauches et 23,4 % (n=22) une hypertrophie concentrique du ventricule gauche. Quatorze patients présentaient une hypertrophie septale. Nous avons retrouvé 18 cas d'accidents vasculaires cérébraux ischémiques et 5 hémorragiques au scanner cérébral. Sur le plan topographique, nous avons observé 43,4 % (n=10) de localisation sylvienne, 17,4% (n=4) de localisation périphérique et 47,8% (n=11) de localisation centrale. Sur 46 patients ayant réalisé un examen du fond d’œil, 4 présentaient une rétinopathie stade III de Kirkendall, 6 un stade II et 10 un stade I.

**Figure 3:**
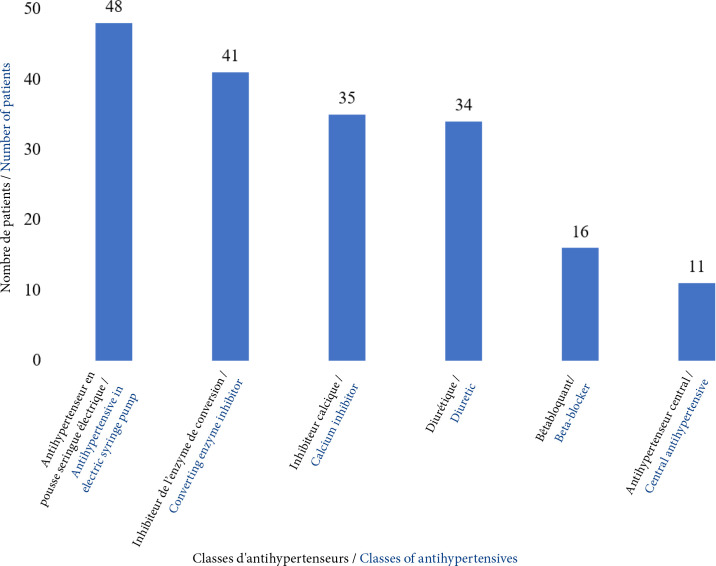
Répartition du traitement des patients à la sortie de l'hospitalisation au CHU-Bogodogo

Sur le plan thérapeutique à l'admission, 51 % des patients ont été mis sous nicardipine au pousse seringue électrique. Les autres antihypertenseurs, à savoir les inhibiteurs de l'enzyme de conversion (captopril), les inhibiteurs calciques (amlodipine), les diurétiques (furosémide) et les bétabloquants (cardensiel) ont été employés respectivement dans 42,7% (n=41), 36,4% (n=35), 35,4% (n= 34) et 16,6% (n=16) des cas. L'alpha-méthyl-dopa a été administré à 11 patients (11,4%). Plusieurs associations médicamenteuses en bi, tri ou quadrithérapie ont été utilisées. Ces traitements ont été ajustés durant l'hospitalisation afin d'obtenir des combinaisons adaptées à la sortie (Figure 3).

## Discussion

La présente étude avait pour objectif d’évaluer l’épidémiologie des UH dans le CHU de Bogodogo durant la période de mars 2017 à décembre 2023. Ses résultats mettent en lumière une prévalence des UH de 3,6 % avec de grandes morbidités telles que les AVC ischémiques, les œdèmes pulmonaires aigus et les insuffisances rénales aiguës, soulignant ainsi leur importance clinique. Le caractère monocentrique et l'absence d'exploration à visée étiologique constituent cependant les principales limites de cette étude. L'aspect innovant de ce travail dans ce nouveau centre, la jeunesse de la population observée et les conditions socioéconomiques difficiles sont les points forts de nos travaux.

La prévalence rapportée dans notre étude pourrait être sous-estimée car notre cible était constituée essentiellement des patients admis dans le service de cardiologie du CHU-B. Notre résultat est inférieur à ceux de Merlo *et al.* et Gombet *et al.* qui ont trouvé respectivement 15 % et 17 % de poussée hypertensive sévère chez une cohorte de 89 patients hypertendus suivis pendant 30 mois [4, 9]. Comparativement aux autres séries, que ce soit dans les pays en développement ou dans les pays développés, seulement moins d'un quart des patients ne se savaient pas hypertendus [6, 20]. Plusieurs hypothèses peuvent expliquer cela. Le manque de moyens financiers pour l'achat des médicaments occasionne l'interruption brutale du traitement antihypertenseur par certains patients à l'origine des poussées hypertensives. En outre, les symptômes de l'HTA correspondent également à ceux de la plupart des maladies tropicales comme la fièvre typhoïde, les fièvres hémorragiques ou le paludisme dont les céphalées, les vertiges et les hémorragies constituent les symptômes majeurs. Ainsi, les patients sont alors traités pour un paludisme par automédication dans un premier temps, et le diagnostic de l'HTA est relayé au second plan lors des crises hypertensives.

Les principaux motifs de consultation faisant découvrir les urgences hypertensives dans notre étude étaient des céphalées (46,8 %), des vertiges (19,1 %) et un déficit moteur (16 %). Plusieurs séries (notamment celles de Zampaglione *et al.* et Guiga *et al.),* rapportent quasiment les mêmes motifs de consultation aux cours des signes d'urgences hypertensives [5, 21].

L’âge moyen dans notre population (50,3 ± 14,6 ans, avec la médiane à 50 ans) est relativement jeune par rapport aux autres séries de la littérature, notamment celle de Guiga *et al.,* qui trouvent un âge moyen de 70 (± 14) ans pour les hommes et 72 (± 19) ans pour les femmes avec un sex-ratio de 0,92 [5].

Cette observation s'inscrit en droite ligne avec celle de Ogah *et al.* qui constate un âge moyen de survenue de l'HTA en Afrique sub-saharienne de 40 ans [11]. Selon certains auteurs, les populations noires, qu'elles résident en Afrique, aux Caraïbes, aux États-Unis ou en Europe, développent une HTA et des lésions d'organes associées à un âge plus jeune. Elles présentent une fréquence plus élevée d'HTA résistante et nocturne et ont un risque plus élevé de maladie rénale chronique, d>accidents vasculaires cérébraux, d'insuffisance cardiaque et de mortalité que les autres groupes humains [1, 16]. Plusieurs théories ont été évoquées pour expliquer ce risque cardiovasculaire accru par de probables différences physiologiques, notamment un système rénine-angiotensine inhibé, l'altération de la gestion rénale du sodium, l>augmentation de la réactivité cardiovasculaire et un vieillissement vasculaire précoce (rigidité des artères de gros calibre) [1, 3, 17].

Dans notre étude, l'hypertension artérielle et le diabète sont les comorbidités prédominantes avec respectivement 80,8 % et 12, 8%. Les principaux facteurs de risque, notamment l'hypertension artérielle, la présence d'une coronaropathie, le nombre élevé d'antihypertenseurs, et surtout le défaut d'observance thérapeutique sont similaires à l’étude de Guiga *et al.* [5].

Une hypertrophie du ventricule gauche (HVG) est notée chez 34,2 % des patients à l’électrocardiogramme et l’échodoppler cardiaque confirme l'HVG chez 22 patients, témoignant ainsi d'une longue période d’évolution d'une HTA bénigne non diagnostiquée. Yameogo *et al.* relevaient une HVG électrique chez tous les patients et une HVG concentrique chez 82,4 % des patients [20]. Cette différence serait due au fait que leur étude a concerné uniquement les patients admis pour super hypertension (pression artérielle systolique ≥ 250 mmHg et/ou une pression artérielle diastolique ≥ 150 mmHg) avec un échantillon beaucoup plus petit.

Les urgences hypertensives vraies représentent trois quarts de notre échantillon et sont dominées par l'accident vasculaire ischémique et l’œdème aigu du poumon à raison de 18 patients chacun. Cette forte représentativité s'explique par la recherche systématique d'atteintes d'organes cibles dans notre étude. Les crises hypertensives constituent généralement les principales circonstances de découverte de l'HTA dans le contexte africain [20]. Les atteintes rénales sont retrouvées chez 15 patients, en accord avec plusieurs études africaines [10, 20]. L'utilisation des produits de la pharmacopée traditionnelle et l'automédication avec risque de toxicité rénale lors des prodromes pourraient expliquer cela dans notre contexte. L’élévation sévère de la pression artérielle (PA), sauf urgence neurovasculaire, requiert sa réduction immédiate pour prévenir ou limiter un dommage viscéral. Dès qu'une surveillance intensive est mise en place, le traitement est administré par voie veineuse directe au pousse seringue électrique, en ajustant précisément le débit en fonction de la PA obtenue [15]. L'objectif recommandé est une réduction de 20 % de la PA moyenne les deux premières heures quelle que soit l'atteinte organique [14].

La Société française de l'hypertension artérielle préconise une combinaison des cinq classes d'antihypertenseurs qui ont démontré une prévention des complications cardiovasculaires chez les hypertendus en absence d'urgence hypertensive vraie [2]. Par ordre d'ancienneté, il s'agit des diurétiques thiazidiques, des bêtabloquants, des inhibiteurs calciques, des inhibiteurs de l'enzyme de conversion et des antagonistes des récepteurs à l'angiotensine 2. Les bêtabloquants apparaissent moins efficaces que les autres classes pour la prévention des accidents vasculaires cérébraux selon la littérature.

La PA a été contrôlée durant l’étude dans seulement 51 % des cas (Tableau I). Nos résultats sont largement supérieurs aux 39 % de contrôle tensionnel retrouvés au Cameroun [10]. Ce meilleur taux de contrôle tensionnel s'expliquerait par l'utilisation des antihypertenseurs par voie parentérale, l'observance thérapeutique et surtout le changement du style de vie des patients en cours d'hospitalisation. Nous n'avons enregistré que deux cas de décès. Yameogo *et al.* avaient obtenu un taux brut de létalité de 4 % personnesmois sur deux ans. Ils notaient que la mortalité chez les patients hypertendus était plus importante lorsqu'il y avait une atteinte rénale associée [20]. Le faible taux de létalité dans notre cohorte pourrait s'expliquer par le traitement actif dès leur admission et surtout l’âge jeune de notre population sans comorbidités majeures.

Les limites de notre étude sont en rapport avec le caractère monocentrique et l'absence d'exploration à visée étiologique.

## Conclusion

Les urgences hypertensives sont relativement fréquentes dans notre contexte. Au CHU de Bogodogo, les atteintes viscérales sont dominées par l'accident vasculaire cérébral ischémique, l’œdème aigu du poumon, et l'insuffisance rénale aiguë. Bien que le pronostic vital soit relativement bon, plusieurs atteintes organiques ont été enregistrées. Leur prise en charge a concerné toutes les classes d'antihypertenseurs, surtout lorsqu'elles étaient administrées au pousse seringue électrique. Une prise systématique de la tension artérielle à toutes les consultations et l’éducation des patients hypertendus sur l'observance au traitement permettraient de réduire les urgences hypertensives.

## Considération éthique

Nous avons obtenu le consentement des sujets concernés. Toutes les dispositions sont prises pour préserver la confidentialité des informations les concernant.

## Contribution des auteurs

NACANABO Wendlassida Martin : rédaction, collecte des données, méthodologie, conceptualisation

SEGHDA Taryètba André Arthur : collecte des données, validation

DAH Djième Claudine : collecte des données SAWADOGO Wendlassida Léa Françoise : collecte des données

SAWADOGO Issa : collecte des données DIMZOURÉ Mohamed Saidou : collecte des données

LOYA Murielle : collecte des données THIOMBIANO Lamoundi Prisca : collecte des données

SAMADOULOUGOU André Koudnoaga : validation, supervision

## Conflit d'intérêt

Les auteurs ne déclarent aucun conflit d'intérêt.
