# Supplementary material for: Hypertensive emergencies at Bogodogo University Hospital, Ouagadougou (Burkina Faso)
Source: Med Trop Sante Int. 2024 Sep 3;4(3):mtsi.v4i3.2024.500. [Article in French] doi: 10.48327/mtsi.v4i3.2024.500 (PMC11809062; doi:10.48327/mtsi.v4i3.2024.500)
Supplement: Related texts

# Research article: Hypertensive emergencies at Bogodogo University Hospital, Ouagadougou (Burkina Faso)

## Introduction

Hypertensive Emergency (HE) is defined as a sudden rise in blood pressure to or above 180/110 mmHg with signs of visceral distress [18, 19]. It requires antihypertensive therapy, but not necessarily blood pressure-lowering therapy [2]. “False hypertensive emergency” or relative emergency is defined as an isolated rise in blood pressure without organ damage [12]. These severe hypertensive attacks are relatively rare, affecting 1-2% of hypertensive patients [8, 12]. The emergency of the situation lies in the identification of acute target organ damage, which requires immediate hospitalization in an appropriate unit [8]. In Burkina Faso, there are no studies on all forms of hypertensive emergencies. The only studies carried out concern malignant hypertension and super hypertension, with a hospital prevalence of 18.4% and 12.9% of hypertensive patients, respectively [13, 20]. The aim of this study was to evaluate the epidemiologic, clinical, paraclinical and therapeutic aspects of hypertensive emergencies in the Cardiology Department of Bogodogo University Hospital (CHU-B).

## Patients and method

This was a descriptive cross-sectional study conducted from March 5, 2017, to December 31, 2023 in the cardiology unit of CHU-B. Patients admitted for hypertensive emergencies were included in the study. The variables collected were:

- Sociodemographic and clinical data, taking into account cardiovascular risk factors, hemodynamic status and signs of visceral distress;
- Electrocardiographic parameters (ventricular hypertrophy, presence of ischemia-injury-necrosis, rhythm or conduction disturbances);
- Echocardiographic and Doppler parameters (state of the right ventricle, dilated or not, presence of thrombus, left ventricular systolic function, impaired or not);
- CT parameters (type and location of cerebral vascular damage);
- Retinal damage classified in three stages according to Kirkendall [7];
- Biological parameters, including proteinuria, glycemia, troponin, lipids, creatinine and azotemia.

Data entry and statistical analysis were performed using Epi-info software. The description of the different variables was done by calculating proportions for qualitative variables and location and dispersion parameters for quantitative variables.

## Results

Of the 2,610 patients admitted to the cardiology unit for all causes, there were 96 cases of hypertensive emergencies, representing a hospitalization frequency of 3.6%. The flow chart of our study is shown in Figure 1.

**Figure 1:** Patient flow chart

The median age of patients was 50 years (first quartile 38 years old and third quartile 60 years old), with extremes ranging from 23 to 83 years. There was a female predominance, with 55 women (58.5%) *versus* 39 men (41.5%). Seventy-six of our patients (80.8%) had a history of hypertension. Headache, dizziness, and motor deficits were the main reasons for consultation in 44, 18, and 15 patients, respectively (Table I).

**Table I:** General characteristics of patients in CHU-B

Variables	Patients (n)	Proportions (%)
Cardiovascular risk factors and co-morbidities		
Hypertension	76	80,8
Sedentary lifestyle	68	72,3
Diabetes	12	12,7
Tobacco	9	9,5
Familial hypertension	7	7,4
Chronic kidney disease	5	5,3
Reasons for consultation		
Headaches	44	46,8
Vertigo	18	19,1
Motor deficit	15	15,9
Tinnitus/phosphenes	9	9,5
Evolutionary aspects		
Median length of hospital stay in days	05	[1;5]
Blood pressure checked at discharge	48	51
Deaths	2	2

True hypertensive emergencies affected 73.4% (n=71) of the patients. True emergencies included ischemic stroke in 18.7% (n=18) of cases, acute pulmonary edema in 18.7% (n=18) and acute renal failure in 15.6% (n=15) of cases. One quarter of the studied group presented a relative emergency. The different types of hypertensive emergencies are summarized in Figure 2.

**Figure 2:** Graphical representation of patients according to type of hypertensive emergency in CHU-B

The electrocardiogram showed atrial and left ventricular hypertrophy in 41.5% and 36.2% of cases, respectively. Four patients had anterior hemiblock and atrial fibrillation was observed in five patients. On transthoracic echocardiography, 12.8% (n=12) had dilated left cavities and 23.4% (n=22) had concentric left ventricular hypertrophy. Fourteen patients were found to have septal hypertrophy. We found 18 cases of ischemic and 5 cases of hemorrhagic stroke on brain CT. Topographically, 43.4% (n=10) were sylvian, 17.4% (n=4) peripheral, and 47.8% (n=11) central. Of the 46 patients who underwent fundus examination, 4 had Kirkendall stage III, 6 stage II and 10 stage I retinopathy.

On admission, 51% of the patients were started on nicardipine with an electric syringe pump. Other antihypertensive agents, namely angiotensine-converting enzyme inhibitors (captopril), calcium channel blockers (amlodipine), diuretics (furosemide), and beta-blockers (cardensiel) were used in 42.7% (n=41), 36.4% (n=35), 35.4% (n=34), and 16.6% (n=16) of the cases, respectively. Alphamethyl-dopa was administered to 11 patients (11.4%). Several drug combinations were used in bi-, tri- or quadruple therapy. These treatments were adjusted during hospitalization to achieve appropriate combinations at discharge (Figure 3).

**Figure 3:** Patients treatments on discharge from CHU-Bogodogo

## Discussion

The aim of the present study was to evaluate the epidemiology of HE in Bogodogo University Hospital during the period from March 2017 to December 2023. The results show a prevalence of HE of 3.6% with major morbidities such as ischemic stroke, acute pulmonary edema and acute renal failure, highlighting its clinical importance. However, the monocentric nature of the study and the lack of etiologic investigation are its main limitations. The innovative aspect of this work in this new center, the youth of the population observed and the difficult socioeconomic conditions are the strengths of our work. The prevalence reported in our study may be underestimated because our target population consisted mainly of patients admitted to the cardiology department of CHU-B. Our result is lower than that reported by Merlo *et al.* and Gombet *et al.* who found 15% and 17%, respectively, of severe hypertensive recurrences in a cohort of 89 hypertensive patients followed for 30 months [4, 9]. Compared to other series, whether in developing or developed countries, only less than a quarter of the patients were unaware that they had hypertension [6, 20]. There are several possible explanations for this. Lack of financial resources to purchase medication causes some patients to stop antihypertensive treatment abruptly, leading to hypertensive relapse. In addition, the symptoms of hypertension are similar to those of most tropical diseases, such as typhoid fever, hemorrhagic fever or malaria, where headache, dizziness and bleeding are the main symptoms. As a result, patients are initially treated for malaria by self-medication, and the diagnosis of hypertension takes a back seat during hypertensive crises. In our study, headache (46.8%), dizziness (19.1%) and motor deficit (16%) were the main reasons for consultation leading to the discovery of hypertensive emergencies. Several series (especially those of Zampaglione *et al.* and Guiga *et al.)* report almost the same reasons for consultation in hypertensive emergencies [5, 21].

The mean age in our population (50.3 ± 14.6 years, with a median of 50 years) is relatively young compared to other series in the literature, especially that of Guiga *et al.* who found a mean age of 70 (± 14) years for men and 72 (± 19) years for women, with a sex ratio of 0.92 [5].

This observation is consistent with that of Ogah *et al*. who found a mean age of onset of hypertension of 40 years in sub-Saharan Africa [11]. According to some authors, black populations, whether living in Africa, the Caribbean, the USA or Europe, develop hypertension and associated organ damage at a younger age. They have a higher incidence of resistant and nocturnal hypertension and a higher risk of chronic kidney disease, stroke, heart failure and mortality than other human groups [1, 16]. Several theories have been proposed to explain this increased cardiovascular risk by probable physiological differences, including an inhibited renin-angiotensin system, impaired renal sodium management, increased cardiovascular reactivity, and premature vascular aging (atherosclerosis) [1, 3, 17].

In our study, hypertension and diabetes mellitus were the predominant comorbidities with 80.8% and 12.8%, respectively. The main risk factors, especially arterial hypertension, the presence of coronary artery disease, a high number of antihypertensive medications and, above all, poor adherence to treatment, are similar to the study by Guiga *et al.* [5].

Left ventricular hypertrophy (LVH) was detected by electrocardiogram in 34.2% of patients, and cardiac echodoppler confirmed LVH in 22 patients, indicating a long evolution of undiagnosed benign hypertension. Yameogo *et al.* found electrical LVH in all patients and concentric LVH in 82.4% [20]. This difference may be due to the fact that their study included only patients admitted for super hypertension (systolic blood pressure ≥ 250 mmHg and/or diastolic blood pressure ≥ 150 mmHg), with a much smaller sample size. True hypertensive emergencies accounted for three quarters of our sample and were dominated by ischemic stroke and acute pulmonary edema with 18 patients each. This high representation is explained by the systematic search for target organ damage in our study. Hypertensive crises are generally the main circumstances for the detection of hypertension in the African context [20]. Renal damage was found in 15 patients, in agreement with several African studies [10, 20]. The use of traditional pharmacopoeia products and self-medication with the risk of renal toxicity during prodromal stages could explain this in our context.

Except in neurovascular emergencies, severe elevations in blood pressure (BP) require immediate reduction to prevent or limit visceral injury. Once intensive monitoring is in place, treatment is administered via direct venous access using an electric syringe pump, with the flow rate precisely adjusted according to the BP achieved [15]. The recommended goal is a 20% reduction in mean blood pressure in the first two hours, regardless of organ involvement [14].

The Société française de l'hypertension artérielle recommends a combination of the five classes of antihypertensive drugs that have been shown to prevent cardiovascular complications in hypertensive patients in the absence of a true hypertensive emergency [2]. These are, in order, thiazide diuretics, beta-blockers, calcium channel blockers, angiotensin-converting enzyme inhibitors, and angiotensin-2 receptor antagonists. According to the literature, beta-blockers are less effective than the other classes in preventing stroke.

Blood pressure was controlled during the study in only 51% of cases (Table I). Our results are much better than the 39% blood pressure control found in Cameroon [10]. This better rate of blood pressure control can be explained by the use of parenteral antihypertensives, compliance with treatment and, above all, changes in patients’ lifestyle during hospitalization. We recorded only two deaths. Yameogo *et al.* found a crude case fatality rate of 4% person-months over two years. They found that mortality in hypertensive patients was higher when renal impairment was present [20]. The low case-fatality rate in our cohort could be explained by the active treatment on admission and especially by the young age of our population without major comorbidities. The limitations of our study are related to its monocentric nature and the lack of etiologic investigations.

## Conclusion

Hypertensive emergencies are relatively common in our context. At Bogodogo University Hospital, visceral damage is dominated by ischemic stroke, acute pulmonary edema and acute renal failure. Although the vital prognosis was quite good, several organ dysfunctions were noted. Treatment included all classes of antihypertensive drugs, especially when administered with an electric syringe pump. Systematic blood pressure measurement at all consultations and education of hypertensive patients about adherence to treatment would help reduce hypertensive emergencies.

## Ethical considerations

Informed consent was obtained from the subjects. All measures were taken to maintain the confidentiality of the information concerning them.

## Authors’ contributions

NACANABO Wendlassida Martin: writing, data collection, methodology, conceptualization SEGHDA Taryètba André Arthur: data collection, validation

DAH Djième Claudine: data collection SAWADOGO Wendlassida Léa Françoise: data collection

SAWADOGO Issa: data collection DIMZOURÉ Mohamed Saidou: data collection LOYA Murielle: data collection THIOMBIANO Lamoundi Prisca: data collection SAMADOULOUGOU André Koudnoaga: validation, supervision

## Declaration of interest

The authors declare no conflict of interests.
